# The experience of diabetic retinopathy patients during hospital-to-home full-cycle care: A qualitative study

**DOI:** 10.1186/s12912-023-01206-y

**Published:** 2023-03-03

**Authors:** Mengyue Zhang, ChunHua Zhang, Chen Chen, Linjie Liu, Youping Liang, YiRong Hong, Yanyan Chen, Yinghui Shi

**Affiliations:** 1 School of Ophthalmology and Optometry, Wenzhou Medical University, 270 West Xueyuan Road, 325027 Wenzhou, Zhejiang China; 2 The Eye Hospital of Wenzhou Medical University, 270 West Xueyuan Road, 325027 Wenzhou, Zhejiang China

**Keywords:** Diabetic retinopathy, Patient experience, Nursing, Qualitative research, Timing it right, Vitrectomy

## Abstract

**Background:**

Diabetic retinopathy (DR) is one of the major blinding eye diseases worldwide. Psychological, emotional and social problems of DR patients are prominent. The aim of this study is to explore the experiences of patients with different phases of DR from hospital to home based on the “Timing It Right” framework, and to provide a reference for formulating corresponding intervention strategies.

**Methods:**

The phenomenological method and semi-structured interviews were used in this study. A total of 40 patients with DR in different phases were recruited from a tertiary eye hospital between April and August 2022. Colaizzi’s analysis method was used to analyse the interview data.

**Results:**

Based on the “Timing It Right” framework, different experiences in five phases of DR before and after Pars Plana Vitrectomy (PPV) were extracted. The patients experienced complicated emotional reactions and inadequate coping skills during the pre-surgery phase, increased uncertainty during the post-surgery phase, insufficient confidence and the decision to change during the discharge preparation phase, eagerness for professional support and moving forward in exploration during the discharge adjustment phase, and courageous acceptance and positive integration during the discharge adaptation phase.

**Conclusion:**

The experiences of DR patients with vitrectomy in different phases of disease are ever-changing, and medical staff should provide personalized support and guidance to help DR patients get through the hard times smoothly and enhance the quality of hospital-family holistic care.

**Supplementary Information:**

The online version contains supplementary material available at 10.1186/s12912-023-01206-y.

## Introduction

Diabetic retinopathy (DR) is one of the most common microvascular complications among diabetics and the main leading cause of blindness in adults [1]. Proliferative diabetic retinopathy (PDR) is one of the most visually impairing complications of DR [2]. According to the latest data, 463 million adults currently have diabetes worldwide, with China ranking first [3]. Approximately 60% of diabetics will develop PDR [4]. The global prevalence of DR is 34.6% among diabetes patients and more than 50% of visual impairment or blindness cases caused by DR come from the Asia-Pacific region [5, 6]. These sobering statistics indicate that DM has emerged as a major global health problem and it is urgent to step up efforts to combat it.

Regular screening and intensive glucose management can effectively delay the progression of DR [5, 7]. However, diabetics in China often have poor awareness of ophthalmic screening and poor self-management ability. Only 39.7% of diabetics have good glycaemic control and meet the recommended target levels [8], and up to 67% of diabetics have PDR at the time of their first ophthalmology visit [9]. The present study deeply explored the feeling of illness and experience of patients with DR using a qualitative research methodology; thus, providing a basis for intervention studies.

### Background

Vitrectomy is one of the mainstays of DR treatment. However, patients are only able to recover or retain partial useful vision after surgery, and most patients continue to have psychological, emotional, and social problems [2, 10]. Meanwhile, visual impairment due to DR has a huge impact on quality of life and can cause concern and emotional distress by limiting mobility, activity, and socialization [11, 12]. According to statistics, almost 25% and 13.5% of patients with DR show symptoms of depression and anxiety, respectively, in China [13]. The psychological, emotional, and social problems are more prominent in patients with PDR [14], which may reduce compliance, reduce the level of blood glucose management, and accelerate disease progression in patients with DR [15]. Therefore, the psychosocial situation of DR patients requires attention to meet their relevant needs.

Until now, many studies have focused on interventions for patients with DR and their effectiveness [16, 17]. Some studies explored the disease-related experiences of DR patients [18, 19]. However, little is known about the full cycle of experiences of DR patients treated by vitrectomy during the hospital-to-home in China. Cameron and Gignac (2008) proposed the theory of “Timing It Right” (TIR) [20], which divided the disease process into five stages: (1) event/diagnosis, (2) stabilization, (3) preparation, (4) implementation and (5) adaptation. The first two phases occur during acute care, the third occurs during acute care and/or inpatient rehabilitation, and the final two phases occur in the hospital-family community. Each stage focuses on information, emotion, tools, and assessment needs, and further emphasizes that patients’ care needs vary with time. Using the TIR theoretical framework and a qualitative approach allow for a better understanding of the experiences and feelings of DR patients from their perspective during disease different stages, thus enabling the needs of DR patients to be more fully responded to.

Therefore, this study aims to explore the experiences and feelings of DR patients during different disease treatment stages through in-depth interviews based on the theory of “Timing It Right”. This is helpful to provide a reference for formulating effective and sustainable hospital-family holistic care intervention programs.

## The study

### Aims

This study was part of a larger research project to develop a hospital-family holistic caring intervention program for patients with DR and discuss the effect of its application on DR patients through a quasi-experimental study.

### Primary objective

The primary objective of this qualitative study was to explore the experiences and feelings of DR patients at different phases and understand their relevant needs.

### Secondary objective

To provide a reference for formulating the intervention program that meets the needs of these patients.

### Design

This qualitative design was adopted using the Descriptive Phenomenology approach. Descriptive phenomenology emphasizes a process of “returning to the thing itself” and gives more attention to the life experience of patients, which helps to explore the experiences and feelings of patients[21]. Since the study focused on exploring the experiences and feelings of patients with DR in different phases, the descriptive phenomenology research methodology was appropriate.

### Participants

Patients with DR were voluntarily recruited from the Fundus Surgical Department, Eye Hospital of Wenzhou Medical University, Zhejiang, China. Purposeful sampling combined with a maximum variation sampling approach was utilized. Participants with different sociodemographic characteristics were chosen when possible.

Recruitment of participants continued until data saturation was reached [22], which occurred with the 7th, 12th, 12th, 11th and 10th participants for each phase respectively; two researchers agreed no new themes were identified from the interview data. The inclusion criteria were (1) met the relevant diagnostic criteria for DR in the Clinical Diagnosis and Treatment Guidelines for DR in China [23], (2) underwent elective vitrectomy, (3) age ≥ 18 years, (4) had normal verbal communication skills and (5) voluntary participation in this study. Exclusion criteria were (1) other eye diseases or a history of ocular trauma, (2) severe cardiovascular disease or serious organic disease, (3) cognitive impairment, mental disorder, or not fully capable of acting and (4) being receiving or having received care interventions for other chronic diseases.

### Phases

Based on the TIR framework, combined with the characteristics of DR and vitrectomy, and consulting with specialists in the Fundus Surgical Department, Endocrinology Department and other related fields, five phases were designated: pre-surgery, post-surgery, discharge preparation, discharge adjustment, and discharge adaptation. The pre-surgical period was from the time the DR patient decided to undergo vitrectomy to the time of surgery and this period usually lasted 3–7 d. The post-surgery phase was the period between the patient’s surgery and post-surgery stabilization. This period usually lasted 1–2 d. The discharge preparation phase was from the time the patient achieved medical criteria for hospital discharge to the time of discharge, and usually lasted 1 d. The discharge adjustment phase was the period from the patient’s discharge to home until 3 months after the vitrectomy. The discharge adaptation phase was the period 3 months to 6 months after the vitrectomy. According to the short hospitalization period and quick turnaround of DR patients undergoing vitrectomy, the post-surgery and discharge preparation phases were the same patient. There was no duplication in the other phases of participants. Each phase was denoted as A, B, C, and D. The staging criterion was based on the most diseased eye.

### Data collection

A semi-structured interview guide (Table 1) was used to collect information through face-to-face interviews. This interview guide was developed by the physicians, nurses, and patients and was based on a literature review and project team discussions. The final interview guide was revised after consultation with qualitative nursing experts and fundus surgical specialists, and pre-interviews with three patients. All the interviews were conducted by a postgraduate nursing student who was trained in qualitative research. A research assistant played an auxiliary role which included recording the interviews.

**Table 1:** Semi-structured interview guide

Semi-structured interview guide
1. Based on your condition, could you tell me about your experience^a^?
2. Based on your condition, could you describe your current mood^b^?
3. What difficulties do you currently have in managing your disease?
4. What help have you received? What help would you like to receive?

a. Including medical experience, surgical experience, and home experience

b. Including when the eye is bleeding, when preparing for surgery, when returning to the hospital ward after surgery, when preparing for discharge, when silicone oil is placed in the eye, when removing the silicone oil from the eye

Patients who were interested in participating in the study and met the inclusion criteria were informed of the purpose and significance of the study and signed an informed consent form. The interview consisted of five phases. The first interview phase occurred during the pre-hospitalization period and was conducted in the outpatient waiting room. The second and third phases were conducted during the inpatient period, the interview location was a quiet place such as a ward or duty room, and the interview time was during non-treatment and patient rest periods. Patients had returned to the community during the latter two phases and when convenient the latter two interviews were conducted in the outpatient waiting room at the time of patient review or were recorded by video. Each person was interviewed one time, and each interview lasted approximately 20–40 min. The entire interview was recorded, and the researcher listened carefully and recorded the interviewees’ expressions, movements, and emotional reactions.

### Ethical consideration

Ethical approval for this study was granted by the ethics committee of the hospital (approval number: 2022-045-K-30-01). Informed consent was provided and obtained from all participants before the study commenced. To protect the privacy of interviewees, the interviews were presented anonymously, and names were replaced with letters.

### Data analysis

The recordings were transcribed word by word within 24 h after the interview. Colaizzi’s seven-step method was used for data analysis [24]: (1) reading all interview materials carefully, (2) extracting and labelling meaningful statements, (3) coding meaningful statements preliminarily, (4) classifying the codes into themes and subthemes, (5) merging the formed themes with the research content and describing them in detail, (6) discussing the structural framework of the experience and the feelings of the patients with DR at different phases and (7) returning the theme to the interviewee for confirmation. Two female researchers independently analysed and coded the original data. In case of disagreements, the group discussed and reached a consensus. The data were analysed using NVivo 12.0.

### Rigour

The trustworthiness of this qualitative study was ensured by maintaining the credibility, dependability, confirmability, and transferability of the data [25, 26]. The study interviewer was a master’s degree student in nursing. The interviewer received systematic qualitative training to master qualitative research methods, was experienced in eye hospital practices, and established a good relationship with the patients before the interviews commenced. This facilitated the acquisition of real information. The researcher maintained a neutral attitude during the interview, did not lead or hint, did not interrupt the interviewee at will, and only asked timely follow-up questions, rhetorical questions, and clarifications until no new information emerged. Therefore, credibility was ensured. The collection, analysis, and interpretation of data were continually reviewed and detailed to ensure its dependability. The data extracted from the survey results were described in detail to achieve confirmability. Regarding transferability, this study described in detail the inclusion criteria, exclusion criteria, and demographic characteristics involved. Simultaneously, the Consolidated Criteria for Reporting Qualitative Research (COREQ) checklist was used to report the findings [27] (See Appendix I for details).

## Findings

A total of 40 respondents were enrolled, and the number of respondents at each phase was 7, 12, 11 and 10, including 25 males and 15 females. The average duration of diabetes was 13 years. The general characteristics of the study respondents are presented in Table 2. The themes in the interviews are presented in Table 3.

**Table 2:** Sample Characteristics(N = 40)

	Characteristic	The pre-surgery phase(n = 7)	The post-surgery phase / The discharge preparation phase (n = 12)	The discharge adjustment phase(n = 11)	The discharge adaptation phase(n = 10)
Gender	Female	3	4	3	5
	Male	4	8	8	5
Age	＜60	6	11	9	7
	≥ 60	1	1	2	3
Education	High school or less	5	11	10	10
	College or higher	2	1	1	0
Employment status	Employed	5	8	6	4
	Unemployed	1	2	2	4
	Retired	1	2	3	2
Residence	City	2	3	5	6
	Rural aress	5	9	6	4
Marital status	Married	5	10	9	8
	Others^a^	2	2	2	2

^a^Including unmarried, divorced, widows, and widowers

**Table 3:** The themes in the interview

Phases	Themes	Sub-themes	Number(%)
The pre-surgery phase	complicated emotional reactions and inadequate coping skills	Fear and Worry	5(71%)
		Regret and Self-blame	3(43%)
		Shadow and Daunt	4(57%)
The post-surgery phase	the increased uncertainty	Physical discomfort after surgery	3(25%)
		Uncertainty about disease prognosis	6(50%)
		Heavy family burden	7(58%)
The discharge preparation phase	the insufficiency of confidence and the decision to change	Lack of self-care confidence	7(58%)
		Deciding to make a change for the eyes	10(83%)
The discharge adjustment phase	eager for professional support and moving forward in exploration	Eager for professional support	8(73%)
		Trouble with low vision and impaired mental health	7(64%)
		Making changes and moving forward in exploration	7(64%)
The discharge adaptation phase	courageous acceptance and the positive integration	Compromise acceptance and positive transformation	8(80%)
		Gratitude and active integration	6(60%)

### The pre-surgery phase: complicated emotional reactions and inadequate coping skills

#### Fear and worry

In DR fundus haemorrhage, the patient’s sudden blurring of vision and the floating of black shadows in front of the eyes cause the patient to experience fear, anxiety, and other negative emotions.

A2: ‘One day I woke up and my eyes suddenly went blind. I didn’t realize the haemorrhage was so severe.’

A5: ‘When I got up to wash my face, I found that the cobwebs inside my eyes had fallen out. I was scared to death.’

Vision loss caused many inconveniences in the working lives of patients with DR, such as difficulties driving, walking, using WeChat, injecting insulin, etc. This intensified patient anxiety.

A3: ‘Last time I almost hit the old lady, so I am afraid to walk alone now.’

A6: ‘I can’t see with my eyes. I can’t do anything.’

#### Regret and self-blame

In the early stages of diabetes, many patients do not know or believe that diabetes can cause diabetic retinopathy, diabetic foot and other related complications, and do not realize the importance of diabetes glucose management. When vision is impaired, patients with DR begin to regret their previous behaviour.

A3: ‘I didn’t know regret until something went wrong with my eyes. I regret that I didn’t look at my eyes earlier.’

A6: ‘It may not be so serious if it is controlled at the beginning.’

A7: ‘Why didn’t I control it before? Why was my mouth so greedy? Why was my self-control so poor?’

#### Shadow and daunt

Some patients with DR have undergone multiple panretinal photocoagulation (PRP) and anti-vascular endothelial growth factor (anti-VEGF) treatments before being treated with vitrectomy.

A1: ‘The two eyes have already been injected several times, two or three times, and the PRP treatment has been done several times.’

The repeated PRP treatments and the pain associated with the PRP usually cast a psychological shadow on DR patients, making them fearful of subsequent treatments.

A2: ‘When I had PRP treatment before, I felt a little scared. The cloth came up in layers and just showed you an eye.’

A5: ‘I’m definitely not doing PRP treatment. The eye is like being gouged out. It’s more painful than giving birth. I give up on the left eye, I’m still left with that one after this one goes blind.’

### The post-surgery phase: the increased uncertainty

#### Physical discomfort after surgery

The treatment of choice for DR is usually vitrectomy followed by insertion of either a gas or silicone oil tamponade. The procedure utilises local anaesthesia. After the procedure, the anaesthetic effect wears off and patients complain of eye pain.

B5: ‘I want to lower my head a bit, my eyes are not very comfortable, my eyes are swollen and painful. The whole procedure is also very painful. I feel like my eye is bursting open.’

B8: ‘The whole eye is going to explode, now the pressure of the eye is so heavy, as if a mountain is pressed, the eye cannot open.’

Surgical trauma and elevated intraocular pressure (IOP) are the main factors causing postoperative pain. Additionally, if gas or silicone oil is filled in the eye, the patient usually needs to maintain the head-down and side-lying positions alternately after surgery. A prolonged prone position will cause pain in the patient’s head, chest, abdomen and extremities, compress the eye orbit, affect blood circulation and aggravate eye swelling [28].

B10: ‘I never thought I would be like this after the surgery. I couldn’t eat, I want to vomit, and my blood pressure is still high. So, I am in a bad mood. I am told to sit during the day and lie on my side at night, I am so tired. My eyes are swollen and cannot be opened.’

#### Uncertainty about disease prognosis

Due to the filling of gas and silicone oil, DR patients will not have a significant change in vision immediately after surgery compared to pre-surgery. Meanwhile, those who have had silicone oil injected need a second surgery to remove the silicone oil. The uncertainty of the time of the second surgery and the uncertainty of the recovery of vision will increase the patient’s uncertainty about their disease prognosis.

B1: ‘I don’t open my eye. I worry whether it can open. I always expect my eyes to recover better… I’m afraid that eye still cannot see after the surgery.’

B5: ‘I don’t know what the condition of my retina is, do you have the report card from the surgery? What is the condition of my eyes now? Are 800 points of laser considered too much? I am worried that I will see less after the surgery.’

B11: ‘Do I get the PRP treatment next or the anti-VEGF treatment? How can I best maintain my vision?’

#### Heavy family burden

The course of the disease with DR is long and the condition often recurs. Patients mostly need a combination of PRP, anti-VEGF and vitrectomy treatments. Treatment requires tremendous energy and financial resources, which puts heavy care and financial burdens on patients and their families.

B3: ‘Last year the doctor said it would take 20,000. I only had more than 10,000 in my pocket, so I walked away. This year the eye is really no good, my brothers and sisters lent a little to me to do surgery.’

B12: ‘The doctor told me to do the other eye as well. I’m not blind. I’m not doing it for now. This surgery costs more than 20,000 yuan, and my salary is only 1000 yuan a month.’

In addition, the social roles of DR patients at this age are more complex, as they are both sons and daughters and parents. As sons and daughters, they need to support the elderly; as parents, they must raise their children. The multiple roles make their caregiving burden heavy and many neglect themselves.

B3: ‘My two couples earn 100,000 yuan in school, the children’s school needs 50,000 yuan, the family also needs to spend money, not much money left in a year.’

B9: ‘Our generation is in the situation of “the elderly above and children below”, we are very tired, so we do not have time to pay attention to our own health. It is too late once we get sick.’

### The discharge preparation phase: the insufficiency of confidence and the decision to change

#### Lack of self-care confidence

Most patients with DR have a short hospitalization period. They lack sufficient knowledge about eye care, handling postoperative complications and so on. As the time of discharge approaches, patients become increasingly worried that they will not be able to take good care of themselves and then develop a sense of helplessness.

B7: ‘I still don’t know how to protect my eyes, for example, I want to eat melon seeds and peas, but I don’t know if I can eat them. Are these peas bad for the eyes? I’m afraid that if I don’t take good care of my eyes, they will bleed again.’

B10: ‘Today the doctor said that the surgery was successful, and that post-operative infection should be prevented. But my body is too sick, my body is not immune, my immunity is not as strong as others, and I am afraid of infection when I go home.’

B12: ‘The hospital in our town does not provide ophthalmology services, what should we do if we have problems after discharge? Can we add a WeChat? We can consult when we encounter problems.’

#### Deciding to make a change for the eyes

After the patients experienced the inconvenience of blurred vision in their work lives and also had a more comprehensive understanding of the cause and treatment of DR, more than half (10/12) of the interviewees said they realized the importance of blood glucose management and decided to make changes.

B6: ‘Before I thought that as long as the hospital was there, I would not be afraid, and I would be able to come to the hospital for treatment. After this surgery, I realize that my previous knowledge of diabetes was very inadequate, and I will put it in my mind in the future.’

B9: ‘I will definitely manage my blood glucose in the future. It comes out of my eyes and directly affects my work, and I’ve got it in mind.’

B11: ‘We must keep our blood glucose under control. If we don’t control it, we may have big problems with our eyes again later. Whether it is high blood glucose or high blood pressure, it may be harmful to the eyes.’

### The discharge adjustment phase: eager for professional support and moving forward in exploration

#### Eager for professional support

DR patients treated with vitrectomy have a long recovery after surgery, while most patients have a short hospitalization period. An inflammatory response such as conjunctival hyperaemia and corneal oedema still occurs within a short period after discharge. Combined with inadequate self-care ability among DR patients, they urgently want to obtain home support services.

C4: ‘I want to listen to online lectures because sometimes there is something uncomfortable in my eyes and I can learn what causes it. …The consultation channels I also need, otherwise, I am panicking when I have a problem. I want someone who knows more about it to help me.’

C11: ‘I felt fine for the first two days after surgery, then I didn’t know what caused my eyes to get red and swollen, and I really wanted to ask the doctor, but I didn’t know who to ask, and I didn’t have the hospital’s phone number, so I stopped the medication.’

C9: ‘I often ask my doctor in charge. We have added WeChat. I ask him any question and he answers me.’

#### Trouble with low vision and impaired mental health

DR patients whose eyes are filled with silicone oil or gas after surgery still have poor vision. It can make the patient puzzled.

C2: ‘After the surgery on this eye, I still couldn’t see clearly, so I wondered why it was the same before and after the surgery.’

C7: ‘My son and my husband say that I can’t see because of the silicone oil, but I don’t know if that’s the reason.’

At the same time, patients who had high expectations of the surgery before the operation and whose vision recovery was not satisfactory after the surgery will feel a greater sense of psychological disparity. They gradually lose their confidence, and their mental health is impaired.

C6: ‘What is the meaning of my life when I can’t see with my eyes.’

C7: ‘I’m so annoyed that the treatment cost so much money and I can’t see so well. I’m really annoyed.’

C8: ‘Even if the silicone oil is removed, my eyes may still be blind…the vision recovery is so different from what the doctor said before the surgery. I can’t accept it at all.’

Patients did not know how to relieve the pain of impaired vision. However, they did not want to impose it on their family either. Some patients balanced their inner helplessness by complaining about their fate.

C6: ‘I don’t know what to do. I don’t know where the eye problem came from. Why God treated me this way.’

#### Making changes and moving forward in exploration

Most of the respondents mentioned the importance of blood glucose management, and the vision change made them more alert. They started to explore the experience of eye protection and blood glucose management for themselves.

C5: ‘I also bought a glucometer and an automated sphygmomanometer, and now I’m eating more regularly, and my eating habits have slowly adjusted.’

C8: ‘Since the surgery, I’ve been drinking less, and I’ve taken care of myself. I usually sleep at 11 pm, and I don’t stay up until 2 or 3 am.’

C10: ‘Do not smoke, do not touch the kitchen fumes. Cannot eat spicy and stimulating food, we must protect the eyes after surgery, do not let the sweat flow into the eyes in summer.’

### The discharge adaptation phase: courageous acceptance and the positive integration

#### Compromise acceptance and positive transformation

The effect of postoperative vision recovery in patients with DR is different. After the second surgery, some DR patients continue to have low vision, and the distress of low vision makes them reach their lowest point emotionally, showing a loss of self-worth and self-denial.

D1: ‘I can’t read, I can’t write, and I can’t enjoy the scenery, so I have no interest in life.’

Patients mostly emerge from their negative emotions over time. They actively adjust their mindset, seek knowledge about ophthalmology and have regular reviews. They are hopeful for the future.

D1: ‘I’m lucky to be alive now.’

D3: ‘You are sick, no one else is to blame. If you can be cured, it’s good, if you can’t be cured, you learn to accept it.’

D4: ‘If you really can’t keep your eyes, you have to face it. I go to the endocrinology every month to draw blood and prescribe medication now. I am relieved when the doctor said it is okay.’

#### Gratitude and active integration

During this period, DR patients gradually change from their previous fear and worry to positive confrontation and gratitude. Most DR patients are trying to reintegrate into their current lives and work.

D5: ‘I was really lucky to meet Director Wu. We are very happy that the surgery was done well. Now I just live my life as usual, watching TV is basically no problem, and I can cook and eat by myself.’

D6: ‘I was a little cranky before, but after the silicone oil was taken, my eyes can see clearly, so I am very happy now. I’m still young. After my eyes rest for some time, I’m going to catch up on my work.’

## Discussion

### Pay attention to patients’ adverse emotions and provide timely information and emotional support

The results of the study show that when DR patients have fundus haemorrhage, patients will experience sudden blurred vision, dark shadows and other subjective feelings, which often make patients feel fear and anxiety. This is consistent with the findings of Shi et al. [18]. At the same time, DR is characterized by complex treatment, long disease course, and unsatisfactory efficacy. Multiple repeated treatments leave a psychological shadow on the patients, which makes them fearful of the next surgical treatment. The emotional response of DR patients not only affects their treatment effect but also influences disease prognosis and accelerates disease progression [29]. Therefore, healthcare professionals should comfort and care for patients, guide them to vent their negative emotions and relieve their fear and anxiety. Additionally, they should communicate with patients promptly, inform them of the causes, risk factors, treatment methods and relevant prognostic information, reduce their fear of follow-up treatment and improve their coping ability.

Additionally, DR patients become concerned about the surgical effect, disease prognosis and outcome in the post-surgery phase, with an increased sense of uncertainty. During the discharge adjustment period, the persistent low vision distress and the discomfort of special body positions make DR patients feel negative emotions such as anxiety and depression. Their mental health is impaired. Some DR patients in the adaptation phase after secondary surgery still have poor postoperative outcomes. They have their hopes dashed, show a loss of confidence in continuing treatment and a gradual loss of interest in life, and reach their lowest point emotionally. However, the psychology of DR patients is closely related to their self-care ability, blood glucose management and disease prognosis [29, 30]. Therefore, we must address the psychology of DR patients, focusing on DR patients with persistent low vision distress. Various methods should be used to alleviate patients’ negative emotions, such as cognitive behavioural therapy to assess and identify patients’ negative thoughts and help them to effectively overcome their negative thoughts [31]; as well as the emotional freedom technique to encourage DR patients to tap on acupoints to quickly release their negative emotions [32]. Simultaneously, it is also possible to hold patient meetings, to use the power of peer support to promote mutual communication with each other, to carry out emotional catharsis, and to relieve their negative emotions [33].

### To stimulate self-efficacy and positive psychological adjustment to improve quality of life

The results of this study showed that patients mostly expressed the importance of their eyes, the inconvenience caused by their limited vision, and their determination to make changes for their eyes in the discharge preparation phase. They planned to regularly review and manage their blood glucose. This high level of self-confidence in DR patients who decide to make changes to maintain their vision is called self-efficacy. Self-efficacy has a beneficial effect on both glycaemic control and quality of life in DR patients [34]. The study also showed that DR patients had a high level of self-efficacy during the discharge preparation phase and were determined to change. They actively explored self-care modalities that were appropriate for them during the adjustment period. However, the long disease course of DM, the severe complications and the complexity of self-management behaviours make patients with DM prone to diminished self-efficacy [35]. Therefore, during the post-discharge, adjustment and adaptation period, healthcare professionals should conduct activities such as continuous empowerment and supportive education for DR patients from the perspective of self-efficacy [36, 37]. The aim is to involve patients in healthcare decision-making, help them to establish correct perceptions, make self-decisions, conduct self-manage and improve their quality of life.

### Provide DR patients with knowledge and skills guidance to enhance their self-care ability through diversified forms of education

This study found that from the discharge preparation period, DR patients questioned their self-care ability and showed a strong desire for DR knowledge and skills guidance. During the adjustment period, some DR patients expressed the futility of seeking help and demonstrated self-care helplessness at home. They hope to receive continuous and professional care guidance through diversified forms of guidance such as WeChat, telephone, and online lectures. Studies have confirmed that DR patients are prone to complications such as bleeding, infection and increased IOP in the early postoperative period. Moreover, improper postoperative care also increases the risk of recurrent retinal detachment, late recurrent vitreous haemorrhage and related secondary surgery [38, 39]. Therefore, attention should be paid to improving the self-care ability of patients. Healthcare professionals should develop appropriate disease knowledge instruction plans for DR patients at different stages of the disease and provide DR knowledge and skills instruction through multiple channels such as oral, written, video, and audio. In the discharge preparation phase, patients should be evaluated using a self-care ability scale and personalized guidance provided based on assessment results to help them make the transition from hospital to home care [40]. Diversified information exchange is carried out after discharge, and multimedia mobile platforms are fully utilized, such as WeChat public platform and WeChat group to push relevant knowledge and to provide online question-and-answer services. At the same time, patients are regularly evaluated and instructed on their self-care ability by combining outpatient diabetes specialists, case management, home visits and remote follow-ups.

### Building a multidisciplinary platform to achieve high-quality continuity of care

This study found that DR patients face many difficulties in-home care, such as basic diabetic care, eye care, and mental adjustments which are aggravated by persistent impaired vision. They are very eager to receive professional help in many ways. The care of patients with DR involves multiple disciplines such as ophthalmology, endocrinology, and nutrition. A previous study implemented multidisciplinary teamwork continuity of care for patients with DR, and it improved their blood glucose levels and quality of life [41]. Therefore, nursing staff should actively play a leading role in building a multidisciplinary team, providing continuity of care services, and discussing and developing vision maintenance, blood glucose management and follow-up treatment plans with ophthalmologists, endocrinologists, optometrists, dieticians and psychological counsellors. At the same time, the active role of specialist nurses is critical. Monthly telephone follow-ups and home visits are conducted by diabetic and ophthalmic specialist nurses to truly develop and implement continuity of care plans. Additionally, multidisciplinary outpatient clinics should be actively created, the construction of the hospital-community-family care model strengthened, and a comprehensive social support system to provide quality and efficient continuity of care services for DR patients established.

### Limitations

This study had several limitations. First, the interviewees were limited to one tertiary teaching hospital for collection and the results may not be generalizable. Further surveys could be selected for sampling in non-tertiary hospitals. Second, this study only interviewed DR patients, and the generalization of the results may not be comprehensive. Follow-up studies are needed to further understand the relevant experiences of healthcare professionals and caregivers of DR patients, to gain a deeper understanding of the needs of DR patients, and to provide a higher quality and more comprehensive basis for the development of relevant nursing intervention programs. Third, considering the short hospitalization period of DR patients in this hospital, the same patients were interviewed in the post-surgery phase and discharge preparation phase, which may cause some recall bias. The results can be supplemented by future studies in wards that do not use the day surgery model.

## Conclusion

Based on the “Timing It Right” framework, this study conducted in-depth interviews with 40 DR patients at different phases. We found that the feelings and experiences of DR patients were dynamic, ranging from complicated emotional reactions and inadequate coping skills during the pre-surgery period, to increased uncertainty in the post-surgery period, reflecting a strong need for emotional and informational support. From a lack of confidence in self-care during the discharge preparation period to a desire for professional support in the discharge adjustment period, reflecting a need for support for continuity of care; during the adaptation period, most patients compromised, accepted, positively transformed, and actively integrated into their current life trajectory. Therefore, nursing staff should provide appropriate psychological support and professional guidance in a phased and planned manner, especially after the patients are discharged from the hospital, to ensure the continuity of care.

## Electronic supplementary material

Below is the link to the electronic supplementary material.

Supplementary Material 1

## Data Availability

The datasets used and analysed during the current study are available from the corresponding authors on reasonable request.

## Abbreviations

**DR**: Diabetic retinopathy
**PDR**: Pars Plana Vitrectomy
**PRP**: Panretinal photocoagulation
**Anti-VEGF**: Anti-vascular endothelial growth factor
